# Contemplated Alterations in the Law of Lunacy

**Published:** 1853-04-01

**Authors:** 


### CONTEMPLATED ALTERATIONS IN THE LAW OF LUNACY.

THERE are *three* bills introduced by Lord St. Leonards, and now before Parliament, in relation to the law of lunacy, viz., 1st, the "Lunacy Regulation Bill;" 2nd, a bill regulating the "Cure and Treatment of Lunatics;" and, 3rd, a bill entitled "Lunatic Asylums." We have only space for short analyses of these bills in our present number; should they receive the sanction of the legislature, we propose in our July number entering more fully into the subject. We shall first consider the "Lunacy Regulation Bill."

This bill is a revision of the law relating to commissions, *de lunatico inquirendo*, and relating to the management of the property of lunatics; and, we think, is generally a very great improvement upon the present laws relating thereto. It consists of 147 clauses!

The first five clauses are repealing and interpretation clauses.

The following nineteen clauses relate to the appointment of officers and the salaries to be paid them. Amongst the officers so appointed are, as at present, three visitors (two medical and one legal). The medical visitors are to be "physicians in actual practice," and it is provided that, "no person shall be appointed a visitor who shall be, *or shall have been within the two years then next preceding*, directly or indirectly interested in the keeping of any house licensed for the reception of insane persons." That a visitor should be called upon at the time of his appointment to make a declaration that he is not, and while he holds his office that he will not become, directly or indirectly interested in any licensed house is a necessary provision, but with all submission, we must think that the words we have given in italics might well be omitted. The patronage being in the hands of the Chancellor, surely his discretion may be trusted to make a proper selection without tying his hands with a limitation, which we think *must* be *unnecessary* in any case, and which it is quite possible *may*, in its working be found impolitic and injurious.

The next eleven clauses relate to the regulation of the fees and charges in proceedings in lunacy.

It is proposed to raise a fund for these purposes by imposing a per-centage or *ad valorem* payment on the clear amount income of all lunatics; the rate of these payments is to be fixed by the Lord Chancellor from time to time, but an account thereof is to be laid before parliament. In the case of small properties the Chancellor is to have the power to exempt the estate *during the lunatic's lifetime, or the continuance of the lunacy*, from the payment of this per-centage; the arrears thereof to be paid after the lunatic's decease, out of the corpus of the estate.

The succeeding seventeen sections regulate the mode of proceeding in all future inquisitions, and involve very extensive and important changes in the law. Inquisitions are to be conducted by the Master only, *without a jury*, excepting where the Master reports that it would be expedient to have a jury, or where the alleged lunatic demands one; and even in the latter case, if upon personal examination the Lord Chancellor is satisfied "that he is not mentally competent to form and express a rational wish for an inquiry before a jury," the Chancellor may refuse to grant a jury. If, however, the alleged lunatic is residing beyond the jurisdiction of the Court, there *must* be a jury.

At present it is very usual for the finding of the jury to have a retrospective effect, carrying the lunacy back to some period previous to the inquiry; in future *unless under a special order*, this is not to be done, but the finding (whether with or without a jury) is to be confined to the question of lunacy *at the time of the inquiry*.

In cases where the inquiry takes place before a Master only, we think it quite right that the inquiry should be so limited; but where there is a jury we very much question the policy of such limitation, as it must inevitably in many cases lead to further and most costly litigation, because in order to invalidate a will or some other important act which may have been done by the lunatic, proceedings will have to be taken before the jury after the lunatic's death, perhaps after a considerable lapse of time, and when it may be extremely difficult, or even impossible, to obtain evidence which might have been easily obtainable at the time of the inquisition.

The visits of the several visitors are to be regulated from time to time by the Lord Chancellor, or by the "Board of Visitors," but each lunatic must be seen by one, at least, of the visitors once, at least, in every year; and the visitors are annually or oftener to report to the Lord Chancellor the results of their visits.

The reports are to be filed and kept secret in their office, and all reports relating to any particular patient shall upon the death of the patient be forthwith destroyed, or in the case of the inquisition being superseded they are to be destroyed within fourteen days of such supersedeas.

The remaining clauses up to the 143rd, relate wholly to the administration of the lunatic's-estate.

The 143rd and three following clauses relate to the "traverse" of a commission. If it is desired to traverse, notice must be given *within three months* after the return of the inquisition, and no person is to be allowed *to "traverse" more than once*. The practical operation of this clause would be to render a person who had the misfortune to be declared on *two* distinct occasions of unsound mind *always a lunatic*. Is it not possible that this alteration of the law may operate very prejudicially and unjustly in many cases?

*Lunatics' Care and Treatment.*—This bill purports to be an amendment of the 8th and 9th Vict., cap. 100. The alterations, although for the most part unimportant, are certainly in some instances improvements.

Section 2,—Provides that, in the case of all future licences granted for the first time, the person licensed shall reside on the premises.

Section 3,—Repeals the 45th, 46th, 47th, 48th, and 49th sections of the 8th and 9th Vict.

Sections 3 and 4,—Provide that, as heretofore, the order and two medical certificates shall be necessary; and that, when it may have happened that only one certificate could be procured previously to admission, the second is to be furnished within *ten* days, instead of, as formerly, within three days.

Section 6,—Provides that any patient who, having been discharged, is still willing to remain, may be retained as a boarder; but it must be for a specific time, and with the assent of the commissioners; and, with the like assent, a relative or friend of a patient may be admitted as a boarder.

Section 7,—Provides that pauper patients shall not be received without an order and one medical certificate.

Section 8,—Provides that the medical man shall specify in his certificate the facts upon which his opinion is founded, *distinguishing facts observed by himself from facts communicated by others.*

Section 9,—Provides that orders and certificates, if informal, may be amended within fourteen days.

Sections 10, 11, 12, and 13,—Specifying who may sign certificates; the form of the "notice of admission," and the form of the "medical visitation book," are nearly identical with the provisions of the existing act.

Section 14,—Dissolves the "private committee;" vesting its powers in the commissioners at large.

Section 15,—Empowers the commissioners, in the case of single patients, to reduce medical visitations to one in three months.

Section 16,—Provides that every medical man visiting, or having the care of a single patient, shall make an annual report to the commissioners.

Section 17,—Empowers the lord chancellor (upon the report of the commissioners) to discharge any single patient detained in an unlicensed house.

Section 18,—Provides that, in asylums containing less than twenty patients, the visitations may be made by *one* (salaried) commissioner instead of two; and that, when two commissioners are required to visit, it shall not be necessary (as at present) that both shall be salaried commissioners.

Section 19,—Provides for the visitation of workhouses.

Section 20,—Repeals the present exemption of Bethlehem Hospital, placing it under the authority of the commissioners.

Section 21,—Is the interpretation clause, and is important principally as defining what is to be understood by the words "physician," "surgeon," and "apothecary." They shall respectively mean "a physician, surgeon, and apothecary, duly authorized or licensed to practise as such, by or as a member of some college, university, company, or institution legally constituted and qualified to grant such authority or licence in some part of the United Kingdom, and being in actual practice as such physician, surgeon, or apothecary."

Section 22,—Incorporates this act with the 8th and 9th Vict., cap. 100.

Section 23,—Provides that this act shall not extend to criminal lunatics.

Section 24,—Provides that the act is to come into operation on the 1st of June next.

The third bill, entitled, "Lunatic Asylums Bill," relates wholly to County and Borough Asylums.

The first clause repeals the acts now in force. The next forty-four clauses relate to the providing asylums and the appointment of visitors. In every county or borough not having an asylum, the Justices of such county or Recorder of such borough are to take measures for the providing one; and if they neglect to do so for one year after the passing of this act, one of the principal Secretaries of State may call upon them to provide an asylum forthwith. It will, we think, be satisfactory to rate-payers to know that in future there will be some check upon the reckless expenditure which has too frequently been perpetrated in the building of asylums. All plans, estimates, and contracts are to be submitted to one of the principal Secretaries of State, and are not to be carried into effect until approved in writing under his hand.

The forty-sixth and six following clauses relate to the mode of raising money for providing asylums.

The fifty-third and twelve following clauses relate to the regulation and management of asylums, and the appointment of officers—chaplain, resident medical officer, clerk, treasurer, &c. The chaplain to be in priest's orders. Patients not belonging to the Church of England may be visited by ministers of their own persuasion. Superannuation allowances, not exceeding two-thirds of salary, may be granted to officers who may be incapacitated by age, sickness, or infirmity, or who may have served twenty years; but in that case they must be not less than sixty years of age.

The sixty-sixth and twenty-two following clauses relate to the visitation, confinement, removal, and discharge of lunatics.

The first of these clauses we certainly commend to the attentive consideration of the members of our profession.

Every pauper lunatic not in an asylum or registered hospital is to be visited once a quarter by the medical officer of the parish or union in which he may reside; and within seven days after the end of each quarter such medical officer is to prepare and sign a list of all such lunatics, stating therein whether, in his opinion, all or any of such lunatics are or are not properly taken care of, and may or may not properly remain out of an asylum; and within the said seven days he is to transmit such list to the clerk of the guardians; and failing to comply with this enactment, such medical officer is to forfeit not exceeding 20*l.* nor less than 2*l.* With such conditions attached, he is to receive for every such medical visit 2*s.* 6*d.*!!

The sixty-eighth clause is one of very great importance. It provides that upon it being made to appear to any justice that any destitute person (although not actually chargeable to any parish) is a lunatic, and wandering at large, such justice is to cause such alleged lunatic to be brought before him, and if, after being examined by a medical man, it is certified that he is a lunatic, the justice is forthwith to give an order, under his hand and seal, for the reception of such destitute person into an asylum.

And upon it being made to appear to any justice, upon the oath of two or more witnesses, "that any person deemed to be a lunatic (*not* being destitute) is wandering at large, and ought to be confined, or that any person, whether destitute or not, is otherwise *not under proper care or control*, or is cruelly treated or neglected by any relative or other person having the care or charge of him so that he is not properly taken care of," such justice may cause such person to be brought before *two* justices; and if it be certified by a physician, surgeon, or apothecary that he is a lunatic, such justices may give an order for his reception into an asylum or licensed house. If, however, any relative or friend wishes to take the lunatic under his own care, he is to be allowed to do so, upon satisfying the justices that the lunatic will be properly taken care of.

The sixty-ninth clause authorizes the justices, *if they shall think fit to do so*, to order the payment of such reasonable remuneration, as to them may seem proper, to any physician, surgeon, or apothecary, who may have been called in, under the preceding clause. Surely it might have been sufficient to leave the *amount* to the discretion of the justices, although it is, of course, very improbable that a case should arise where the justices should *not* see fit to order *some* remuneration. We think we are justified in demanding, in the name of the profession, that the clause should be made imperative, instead of permissive.

Excepting as regards the cases just referred to, the provisions as to the "order" and "certificates" are identical with the existing law.

As in the case of private patients, the medical man is to distinguish in his certificate "facts" observed by himself from "facts" communicated by others.

Two visitors may order a pauper confined in any other asylum to be removed into the asylum for the county or borough to which the lunatic belongs, and, *vice versâ*, they may, in order to make room for curable patients, order the removal of an incurable patient from the county asylum to some other; but, excepting under such circumstances, and with the special endorsement of a visitor, pauper patients are not in future to be received into any other than the asylum of the county or borough to which they may belong.

Three visitors may, with the advice of the medical officer of the asylum, discharge any patient, whether recovered or not; and visitors may also order any pauper lunatic to be delivered over to the custody and care of any relative or friend, upon such person undertaking that the patient be properly taken care of, and shall be no longer chargeable to any county, union, or parish.

The 79th clause enacts that, "It shall be lawful for the Commissioners in Lunacy, or any two of them, by writing under their hands and seals to order and direct the removal of any lunatic from any asylum, registered hospital, or licensed house, to any other asylum, registered hospital, or licensed house." This clause is copied from the 8th & 9th Vict. cap. 126; but there can be no doubt that as it stood in that act it was supposed to relate only to the cases of pauper patients, and that the omission of the little word "pauper" must have been accidental. It however is clear that in the present instance the omission is intentional; and in order to make it apply to private patients, it is immediately succeeded by three clauses copied from the 8th & 9th Vict. cap. 100, into one of which a parenthetical passage has been introduced referring to this clause.

Bearing in mind, that the 8th & 9th Victoria is *actually under revision at the present time concurrently with this bill*, we are at a loss to understand why, if it is proper to give this power to the Commissioners, it should not be given under *that* act—the act which specially relates to private patients—instead of effecting it by inserting a clause in another act—an act *relating exclusively to county asylums.*

The 83rd clause enacts, that orders and certificates, if informal or incorrect, may be amended within fourteen days.

Then follow the same provisions as to the books to be kept and entries to be made as those in force at present.

The 89th and 27 following clauses relate to the expense of maintenance, removal, &c., and are almost identical with the provisions now in force.

The remaining clauses are "Miscellaneous," and are, also, for the most part, the same as those now in force. The 117th makes it a misdemeanour if any physician, surgeon, or apothecary, shall untruly state anything in his certificate; and, also, if any person shall sign a certificate as a physician, surgeon, or apothecary, not being so; and the "interpretation" clause says, that "physician," "surgeon," and "apothecary" shall respectively mean a physician, surgeon, or apothecary duly authorized or licensed to practise as such by or as a member of some college, university, company, or institution legally established and qualified to grant such authority or licence in some part of the United Kingdom, and being in actual practice as a physician, surgeon, or apothecary.

Query—Why should he be "in actual practice?"

We append to the preceding short abstract of two of the new bills, the following remarks of a non-professional contemporary. We do so for the purpose of placing before our readers the views of intelligent critics out of our own ranks. It is prudent that we should make ourselves acquainted with the sentiments of the public as well as the professional press, upon a question of practical reform, in which all classes of society are deeply interested. We now quote from the *Spectator* of March 12th.

"If the three Lunacy Bills brought in by Lord St. Leonards were at this moment law, a great amount of risk, expense, trouble, and pain, would be saved to numbers of people. On a first perusal of the three bills, they appear to us to meet most of the demands for improvement which recent experience has suggested. The smallest one, which is the most urgent, relates to the care and treatment of lunatics; the second, to the management of lunatic asylums; the third, to the regulation of lunacy business in Chancery. The first and second have a very essential connexion, although it was as well perhaps to separate them; since their provisions ought to be brought under the notice of totally distinct classes of people.

"By the Lunatics' Care and Treatment Bill, new securities are given to the subject against being arrested on the pretext of lunacy, without the necessary checks in the way of medical examination, authorized certificates, visitation by public functionaries, and stated revision by the highest authorities. New forms are given, to be filled up by asylum-keepers, visitors, and medical practitioners: it is a valuable provision that the facts indicating insanity are required to be set forth in the medical certificate. In short, such complete provisions are made for securing a practical knowledge respecting the lunatic, at every stage in his transfer from one hand to another, and during his residence in the asylum, that the abuses which have been notorious since the question was first investigated in 1816 would under this bill become nearly impossible.

"The Lunatic Asylums Bill comprises one hundred and thirty-one clauses, arranged in groups,—as to the providing of asylums and the appointment of local functionaries; as to the raising of monies; as to the regulation and management of asylums; as to the visitation, confinement, removal, and charges of lunatics; as to the expenses of maintenance, &c., of pauper lunatics; with some others. Every county and borough is required to provide a proper asylum for lunatics; unions for the purpose, as in the existing case of Bedford, and of neighbouring counties, being permitted; boroughs below a certain size are to be considered merged in the county; and the Secretary of State is authorized to require the justices of any place to provide a proper asylum, with powers for taking land, &c., and raising money. The asylum is to be visited once in every three months at least. Lunatics are to be sent to asylums in all but specified necessary cases; a provision which will abolish the abuse of detaining them in workhouses, or places even less fitted for the purpose. Ample provisions are made for appeal against detention of lunatics, and a considerable portion of the bill is devoted to provisions for checking needless expenses either to the lunatic or to his friends. A very important clause is the sixty-eighth, which empowers certain parish-officers, upon information, to take any person who is said to be a lunatic, whether destitute or not, before the Justices of the Peace, and to pursue other steps for his proper examination, in order, should he prove lunatic, that he may be kept in proper custody; the friends being permitted to provide for his safe keeping, if they satisfy the Justices that they have the power and the intention so to do. The parish-officers are required to take these steps under a heavy penalty for neglect. If this provision had been in force, it is probable that Pate would not have committed the outrage on the Queen which shocked the country some years ago.

"The Lunacy Regulation Bill is not less important, although it may be less urgently needed. Its general character is, to consolidate the jurisdiction of lunacy business, for the management of property, in two Masters, under whom there are to be a Registrar, and also two medical Visitors, and one legal Visitor appointed by the Lord Chancellor; the Master, the Registrar, and the Visitors, to form a Lunacy 'Board.' In most cases, we infer, the business of inquisition will be carried on by one of the Masters; the lunatic having the power, if the Lord Chancellor be satisfied of his possessing sufficient mental intelligence for holding an opinion, to demand an inquiry before a jury. The Lord Chancellor is also empowered to order the appointment of a jury. As in the previous bill, considerable pains are bestowed upon checking expense, by transacting a portion of the business in chambers, by disallowing useless objections, by discouraging useless citations or diffuseness in documents, and by other means.

In this, as well as in the previous bills, much consideration is shown for the poorer class of lunatics; for instance, where the property is small, it is exempted, more or less, if not entirely, from expenses; and it may be applied to the lunatic's maintenance. Upon the whole, the character of the measure may be described as one for consolidating the law and regulations upon the subject; bringing them all under the direct supervision of the Lord Chancellor; introducing uniformity into the practice; with checks against abuse, and very great checks against needless expense and waste of property.

"The bills would effect such valuable and extensive improvements, that we could almost desire to see them passed exactly as they are, even upon the understanding that new steps must immediately be taken to secure further improvements. Valuable as the bills are, they do not include all that is wanted, nor even all that is *urgently* wanted; yet we mistrust the dilatory and mutilating process of any Select Committee. Not that the further improvements do not suggest themselves readily. We have the very greatest doubts, for example, whether the amount of visiting required by any one of these bills is sufficient. Once in three months is not enough to secure the individual lunatic against an intolerable amount of suffering. Nay, three months is sufficient time to manufacture a lunatic out of an excitable man unjustly imprisoned.

"We might dismiss the doubt, whether the custody of lunatics ought, under any circumstances, to be licensed as a matter of profit. Although to first-rate minds a truly humane system is the one dictated by self-interest as well as duty —although *we are convinced that the most perfect plan of treatment will be that secured in the very best private asylums*—yet we are equally sure, that below that topmost grade, the temptations which make the interest of the keeper adverse to the interest of the lunatic begin to prevail in an accelerating ratio. It is clear from disclosures which have been made quite recently,* that there is a class of minds naturally inclined to an ugly profession, which can only be kept in order by unexpected visits and the force of penalties; and to such minds we do not believe that the helpless can be safely intrusted. We admit, however, that a very law-reformer would naturally hesitate to take a step to abolish the existing interests in private asylums; and we recognise the force of the provisions in the Lunatic Asylums Bill imposing very severe penalties to be recovered summarily, for the offence of striking or ill-treating a lunatic.

"There are other questions also of first-rate importance which lie *between* the measures now before Parliament. For example, there is a class of persons in this country suffering under a kind of grievance which falsifies the maxim of English law "that there is no wrong without redress." According to the general acceptation of law, the lunatic is civilly dead: he can contract no obligations; can perform no civil act; cannot present himself in any capacity; is dead, in short, to all persons except to one—his consort. The incurable lunatic maintains the semblance of a conjugal relation only to prevent the reality. Even to the consort, in all but a few very peculiar and exceptional cases, the incurable lunatic is dead in everything except a mockery of union. It is needless to point out the evil consequences flowing from bondage to a consort who has no practical existence in the world: it is a species of celibacy recognised by our system in no other form. The objection against the dissolution of a marriage, that the lunatic might recover, is one which applies to very few of the cases, and that only in a very doubtful degree. It is to be met also by this remark, that after a consort has been in keeping for a lengthened period, with all but the certainty of hopeless insanity, a renewal of the union can only be effected at the risk of the most calamitous results to the subsequent progeny. On every ground, therefore, a motion for a dissolution of the marriage, *when sought*, ought to be granted on proof of the incurable lunacy. The want of that release imposes upon numbers in this country the cruelty which renders proverbially infamous

* Bethlem Hospital.

the name of Mezentius. We believe that this just principle is recognised by more than one of the United States of America; certainly it is so in the State of New York. But the Lunacy Bills of Lord St. Leonards do not enter into this branch of the question; and we see no intimation that the Divorce Commissioners have yet entered upon the subject of lunacy. If, however, the lunacy laws are to be thoroughly revised, glaring wrongs of this kind would not be suffered to remain; and therefore is it that, while we desire the prompt enactment of the bills as a whole, we anticipate a sequel to supply omissions."

In reference to the same important question, the *Globe* of March 22nd, contains the following just remarks:—

"It is correctly said, that that position violates the soundest principles of law. It is quite inconsistent with the rest of a law which regards the lunatic as dead in effect. The law provides for the management of his property, lest it be wasted for his descendants; the law provides for the performance of his duties as a trustee or guardian; the law releases compacts made with him in which he cannot fulfil his part; but a singular exception is made in the case of that relation in life where death alone is sufficient to free the other party to the bond. If the husband or wife become insane the partner of that afflicted person is still restrained by the bond; although in that particular case, the practical violation of the compact is more manifest and grievous than in almost any other conceivable case. The person wedded to one who is incurably mad, is to all intents and purposes without a husband or a wife, and is as effectually reduced to widowhood as if actual death had supervened; and yet the disabling liabilities of wedlock remain. A person in a state of widowhood by an ordinary death can marry again; a person in a state of widowhood by the civil death of a lunatic must remain in that state of widowhood without release. The consequences of that disability are too apparent to require specification; and although the public at large may not take much interest in these purely personal misfortunes, still there are many upon whom the disability presses with the most painful infliction. There is no case of divorce more clamorous for a just adjudication than one of this kind. It has indeed so pressed itself upon the attention of the Commissioners of Divorce, that they have put forth a series of practical suggestions in the appendix to their first report, just issued; thus challenging attention for the subject, as if desirous that public opinion should be prepared for grappling with it."

